# Immunological and virological triggers of type 1 diabetes: insights and implications

**DOI:** 10.3389/fimmu.2023.1326711

**Published:** 2024-01-04

**Authors:** Joana R. N. Lemos, Khemraj Hirani, Matthias von Herrath

**Affiliations:** ^1^ Diabetes Research Institute (DRI), University of Miami Miller School of Medicine, Miami, FL, United States; ^2^ Division of Endocrine, Diabetes, and Metabolism, Department of Medicine, University of Miami Miller School of Medicine, Miami, FL, United States; ^3^ Global Chief Medical Office, Novo Nordisk A/S, Søborg, Denmark

**Keywords:** type 1 diabetes, viral infection, autoimmunity, enterovirus, COVID-19, environmental factors

## Abstract

Type 1 diabetes (T1D) is caused by an autoimmune process which culminates in the destruction of insulin-producing beta cells in the pancreas. It is widely believed that a complex and multifactorial interplay between genetic and environmental factors, such as viruses, play a crucial role in the development of the disease. Research over the past few decades has shown that there is not one single viral culprit, nor one single genetic pathway, causing the disease. Rather, viral infections, most notably enteroviruses (EV), appear to accelerate the autoimmune process leading to T1D and are often seen as a precipitator of clinical diagnosis. In support of this hypothesis, the use of anti-viral drugs has recently shown efficacy in preserving beta cell function after onset of diabetes. In this review, we will discuss the various pathways that viral infections utilize to accelerate the development of T1D. There are three key mechanisms linking viral infections to beta-cell death: One is modulated by the direct infection of islets by viruses, resulting in their impaired function, another occurs in a more indirect fashion, by modulating the immune system, and the third is caused by heightened stress on the beta-cell by interferon-mediated increase of insulin resistance. The first two aspects are surprisingly difficult to study, in the case of the former, because there are still many questions about how viruses might persist for longer time periods. In the latter, indirect/immune case, viruses might impact immunity as a hit-and-run scenario, meaning that many or all direct viral footprints quickly vanish, while changes imprinted upon the immune system and the anti-islet autoimmune response persist. Given the fact that viruses are often associated with the precipitation of clinical autoimmunity, there are concerns regarding the impact of the recent global coronavirus-2019 (COVID-19) pandemic on the development of autoimmune disease. The long-term effects of COVID-19 infection on T1D will therefore be discussed, including the increased development of new cases of T1D. Understanding the interplay between viral infections and autoimmunity is crucial for advancing our knowledge in this field and developing targeted therapeutic interventions. In this review we will examine the intricate relationship between viral infections and autoimmunity and discuss potential considerations for prevention and treatment strategies.

## 1 Introduction

Viral infections may be a principal trigger for immune system intolerance towards self-antigens, with, genetic factors and immunological fitness of the host determining susceptibility (1). Such immunological autoreactivity may lead to prevalent and debilitating autoimmune conditions such as rheumatoid arthritis (RA), neurodegenerative disorders, and type 1 diabetes (T1D). Viral infections impact many components of the immune system, and the complexity of these interactions contributes to our nominal understanding of why and how such infections may lead to autoimmunity. Therefore, whilst the notion of viral infections as an accelerator or trigger for autoimmunity is a long-standing theory that has been confirmed for some clinical cases, it often remains purely a hypothesis because many gaps in our knowledge of this subject remain.

T1D is a chronic autoimmune disease characterized by the selective destruction of insulin-producing beta-cells in the pancreas. The etiology of T1D is multifactorial and involves a complex interplay between genetic and environmental factors. One key genetic risk factor for T1D is the presence of specific human leukocyte antigen (HLA) alleles, such as HLA-DR3 and HLA-DR4, which are associated with an increased risk of developing T1D (2). Environmental factors that have been implicated in the development of T1D include viral infections, such as EVs, rotaviruses, and herpes viruses such as cytomegalovirus (3–5), as well as dietary factors, such as the early introduction of gluten (6). Mechanistically, T1D is characterized by the activation of autoreactive T cells (the majority of these being CD8^+^) that recognize and attack beta-cells in the pancreas, leading to their destruction and subsequent insulin deficiency (7). This process may be triggered by the presence of viral antigens that share structural similarities with beta cell antigens, leading to molecular mimicry and the activation of autoreactive T cells (8). Other mechanisms that have been proposed to contribute to T1D pathogenesis include the activation of autoreactive T cells via bystander activation or epitope spreading during inflammation (9), as well as the infiltration of the pancreas by other pro-inflammatory immune cells, which produce cytokines such as interferons or tumor necrosis factor (TNF), that could contribute to beta cell destruction(10) (**Table 1**).

**Table 1:** Proposed underlying mechanisms for the potential role of viral infections in the development of autoimmunity with special emphasis on T1D.

Mechanism	Description	Characteristics	T1D Relevance	References
Checkpoints Preventing Autoimmunity	Autoimmunity arises due to a loss of self-tolerance. The immune system begins to attack its own cells, causing dysfunction or destruction.	Circulating islet autoantibodies often precede the appearance of overt symptoms. Insulin autoantibodies are usually the first to appear.	At the onset of T1D, 95% of patients have antibodies to at least one beta cell protein, such as IA-2, GAD-65, or ZnT8. Age, sex, genetics, and environmental exposure are additional susceptibility factors.	Bender et al. (7); Wenzlau et al. (11); Shichkin and Antica (12), Yu et al. (13); Castañeda et al. (14); Sharma and Rudra (15); Ihantola et al. (16); Rodriguez-Calvo et al. (17)
Molecular Mimicry	Viral proteins may structurally resemble host proteins. This can confuse the immune system, causing it to attack both the viral and host cells.	May involve specific viral strains that share structural similarities with islet cell proteins.	Increases the risk of immune system misidentifying pancreatic islet cells as foreign, triggering T1D.	Fujinami et al. (18); Gauntt et al. (19); Vreugdenhil et al. (20); Wucherpfennig and Strominger (21); Zhao et al. (22); Coppieters et al. (23)
Bystander Activation	Viral infection causes activation of immune cells near infected cells, leading to collateral damage to nearby healthy tissues.	Typically involves T cells and may cause localized inflammation.	May lead to activation of autoreactive T cells against pancreatic islet cells, contributing to the onset or progression of T1D.	Shim et al. (9); Christoffersson et al. (24); Smatti et al. (25)
Epitope Spreading to Neoantigens and Cryptic Antigens	After the initial immune activation, the response can broaden to include other antigens not initially targeted.	Often involves secondary or tertiary immune responses, which may be stronger and more specific.	Can widen the autoimmune attack to involve multiple components of islet cells, worsening T1D.	Smatti et al. (25); Quintana et al. (26); Miller et al. (27); Tuohy and Kinkel (28); von Herrath et al. (29)
Persistent Infections	Chronic viral infections can keep the immune system continuously activated, which increases the risk of autoimmunity.	May involve viral latency or periodic reactivation.	Sustained immune activation can enhance susceptibility to T1D by maintaining the autoimmune attack on islet cells.	Op de Beeck and Eizirik (30) Faulkner et al. (31), Nekoua et al. (32)
Mechanisms of Viral-induced Insulin Resistance	Certain viruses can interfere with insulin signaling pathways, inducing insulin resistance.	May involve cytokine release and inflammatory responses that disrupt insulin signaling.	Insulin resistance can further complicate glycemic control in T1D, requiring adjustments in treatment strategies.	McGillicuddy et al. (33); Wilkin (34); Subauste et al. (35); Brooks-Worrell et al. (36)

In this article, we will discuss the potential role for viral infections on the development of autoimmunity, by critically examining the current evidence for the proposed underlying mechanisms. Whilst the review is focused on evidence relating to T1D, most of the principles and mechanisms could also apply to other autoimmune diseases. With the COVID-19 pandemic and subsequent observed increase of T1D and T2D cases following infection as an acute backdrop, we will also highlight investigations studying whether this serious and widespread viral infection could lead to a peak in the incidence of autoimmune illnesses. Towards the end of the review, we provide perspectives on current knowledge gaps and suggest how best to move the field forward with clinical investigations, which may potentially pave the way towards interventions which can prevent or reverse virally-induced autoimmunity.

## 2 Mechanisms

### 2.1 Checkpoints that normally prevent autoimmunity

Autoimmunity is the result of a loss of self-tolerance in the immune system, which then begins to attack its own cells or organs, resulting in their dysfunction or destruction. The appearance of circulating islet autoantibodies typically precedes the development of overt disease symptoms by some years. Insulin autoantibodies are frequently the first to emerge in young children, followed sequentially by any of the others (islet antigen 2 (IA-2), glutamic acid decarboxylase 65 (GAD-65) and zinc transporter 8 (ZnT8). At clinical onset, 95% of patients have antibodies to at least one of these beta cell proteins (11). Several factors play a part in determining the susceptibility of an individual towards the loss of immunologic self-tolerance, including age, sex, genetics, and specific environmental exposure. There are three major levels of security (‘checkpoints’) that prevent autoimmunity from becoming deleterious under normal circumstances. The first checkpoint occurs in the thymus, where the adaptive immune cell repertoire is shaped during its development. Traditionally, this process was believed to eliminate a significant number of cells with receptors that have high affinity for autoantigens (12). However, other authors suggest that the role of thymic deletion may be less significant than previously thought. It was shown an unexpected abundance of self-specific CD8+ T cells in the peripheral blood of healthy adults (37). This challenges the traditional view that thymic deletion is the primary mechanism for eliminating self-reactive T cells. Instead, it indicates a more nuanced mechanism where self-specific T cells are maintained in an anergic state in the periphery, rather than being predominantly deleted in the thymus. Defects in thymic selection can thus lead to increased autoimmunity (14), but the presence of these self-specific cells suggests additional layers of immune regulation.

Secondly, after passing through the thymus, immune homeostasis in the periphery is maintained by a pool of regulatory cells (Treg) (15). Such cells, in many cases, are characterized by low rates of proliferation, and expression of the IL-2 receptor and transcription factor FoxP3. Although earlier studies, such as those by Luczyński et al. (38) and Zahran et al. (39), have observed changes in Treg populations in children with newly diagnosed diabetes, it is increasingly recognized that the core issue lies in Treg dysfunction (13). This dysfunction is crucial in the progression of the disease, as it leads to inadequate suppression of CD4+ effector T cells. A key feature of this impaired regulatory mechanism is the STAT3-dependent resistance of Teffs to Treg-mediated control, a process surprisingly independent of IL-6 signaling pathways. These insights not only redefine our understanding of T1D but also open new possibilities for targeted immune therapy strategies (16). In animal models, the progression of T1D is influenced by experimental depletion or genetic deficiency of Treg (40). Anomalies in the structure of the IL-2 receptor have been noted in T1D, and the disruption of IL-2 synthesis by effector T cells has been proposed as a contributing factor to T1D development. The third checkpoint, for organ specific autoimmune diseases such as T1D, is maintenance of tolerance, or the camouflage of critically important cell types such as insulin-producing beta cells from immune recognition, locally at the target organ. The integrity of this third checkpoint may be disturbed by external factors such as local infections, disruption of natural barriers (skin, mucosa) or through changes in innervation. This is a process that has historically been under-appreciated but is probably of high importance, because it has become increasingly clear that most of us harbor significant numbers of auto-reactive T cells within our peripheral lymphoid organs and at target sites such as the pancreas (7, 17).

Amongst the environmental factors implicated as triggers for the development of autoimmunity, the exposure to pathogens such as viruses has been well-studied. There are several mechanisms by which viruses are thought to contribute to the development of autoimmunity: molecular mimicry, bystander activation, epitope spreading and unmasking of cryptic antigens, direct infection/persistence in the target organ and (in the case of T1D) systemic increase of insulin resistance through inflammation. The relative importance of these mechanisms varies across each autoimmune condition and has become clearer over the past decades – we will discuss each of the mechanisms and provide context based on more recent discoveries.

### 2.2 Molecular mimicry

Molecular mimicry which was first described in 1964 (41), refers to a phenomenon whereby viral or bacterial antigens share structural similarities with self-antigens, leading to the activation of autoreactive immune cells and the purported development of autoimmune diseases. Thus, molecular mimicry could play a role in the development of autoimmune diseases by triggering a cross-reactive immune response against self-antigens. When the immune system mounts a response against a pathogen with a molecular structure similar to that of a self-antigen, the immune response can also target that particular self-antigen, leading to a specific autoimmune reaction. For example, in rheumatic fever, an infection with the bacterium *Streptococcus pyogenes* can lead to the production of antibodies that cross-react with cardiac myosin, a protein found in the heart muscle. This can lead to damage to the heart tissue and the development of rheumatic heart disease (42). Similarly, in multiple sclerosis (MS), it is thought that the presence of a viral protein with a molecular structure that is similar to that of myelin (the protective coating around nerve fibers), such as the Epstein-Barr virus (EBV) protein may lead to a cross-reactive immune response towards myelin, resulting in damage to the nervous system and the development of MS following exposure to this virus (43).

In T1D, molecular mimicry, has also been proposed as a potential mechanism by which viral infections may trigger autoimmunity against the beta cells. Numerous studies have provided support for the role of molecular mimicry in T1D, including studies demonstrating the presence of viral antigens in the pancreas of patients with T1D and the ability of viral antigens to induce autoimmune responses in animal models (18–22). Proteins in viruses like Coxsackievirus, cytomegalovirus, enteroviruses, and rotavirus can mimic human antigens such as GAD65 and IA-2. This mimicry leads to cross-reactive immune responses, where the body’s immune system mistakenly targets its own cells, potentially contributing to the development of T1D (23).

Inflammation induced by exposure to a foreign antigen can lead to autoimmunity from cross-reactive epitopes. These epitopes are segments of foreign antigens which, when presented to either T or B cells in the context of the MHC, can activate CD4+ or CD8+ T cells. The induction of the immune response and subsequent proinflammatory cytokine release is critical for clearance of a virus or bacteria. However, a sustained proinflammatory response against specific host tissues can occur when there is sequence or structural homology between foreign antigens and self-antigens, termed molecular mimicry. Molecular mimicry proposes the activation of auto-aggressive T cells in T1D as the result of a virus carrying an epitope that strongly resembles certain structures on the beta cells, and which consequently induces a cross-reactive autoimmune response that eliminates not only the infecting virus but also pancreatic beta cellsAlthough, while it is possible to trigger T1D via molecular mimicry in engineered animal models (44), it has been difficult to prove that cross-reactivities play any role in human T1D - the reason is that one would have to induce tolerance to such cross-reactive epitopes, which to date has not been clinically possible. The second issue is that animal models have shown that molecular mimicry is unlikely to be acting on its own in otherwise non-autoimmune prone hosts, meaning that it is more likely that cross-reactivities merely push an already primed system over the brink, leading to clinical diagnosis (45). While the concept of molecular mimicry has been an important area of investigation in T1D research in recent years, proof that cross-reactivities play any role in triggering or accelerating autoimmunity will have to await the capability to induce epitope/antigen specific tolerance in humans.

### 2.3 Bystander activation

The ‘bystander activation’ hypothesis proposes that immune cells can be activated in response to a nearby inflammatory signal, even if the immune cell does not directly recognize a specific antigen. Mostly this occurs via the effects of locally-secreted cytokines such as IL-2 and innate immune activators. While it is known that inflammatory pathways can ‘license’ or activate antigen presenting cells (APCs) and that inflammation can activate immune cells through various pathways including TLRs, the term ‘bystander’ is probably the wrong term based on our current understanding for the following reasons: Firstly, if there are direct pathways activating cells, this is not a bystander phenomenon but instead a directional response and secondly, the evidence that adaptive immune cells that do not recognize antigens within an inflammatory lesion can erroneously be activated is non-existent. Instead, studies have shown that T cells which do not recognize antigens function as regulators by dampening the inflammation (24). This also makes much more sense in limiting excessive immune pathology and inflammation: If there are no driving antigens present, the immune response shuts down rather than propagating itself. The problem of autoimmunity is actually different: here self-antigens have inadvertently become driving antigens. Thus, we feel that bystander non-antigenic phenomena are not highly likely to contribute to the development of autoimmunity.

### 2.4 Epitope spreading to neoantigens and otherwise hidden (‘cryptic’) antigens

Epitope spreading is a process in which an immune response against one epitope (antibody binding site on a protein) leads to the recognition and response against additional epitopes. This phenomenon can occur during the progression of an autoimmune disease, where the initial immune response may be directed against a specific epitope, but as the disease progresses, the immune system may begin to recognize and respond to other epitopes on the same or different proteins. It has elegantly been shown to cause relapsing/remitting cycles of disease in the encephalomyelitis model for MS (46) – the new antigens might also be post-translationally modified antigens or so-called ‘cryptic’ antigens that are not usually displayed in the context of MHC on the surface of cells and are only revealed during inflammatory responses. In this case, incomplete thymic tolerance to such antigens might aggravate the problem, as more responder T and B cells might be present in the periphery.

Overall, the phenomenon of epitope spreading could be an important process in the development and progression of autoimmune diseases and may contribute to the broadening of the immune response against self-antigens. This process has been implicated in the development and progression of autoimmune diseases such as MS, RA, and systemic lupus erythematosus (SLE). Studies have shown that in many autoimmune diseases, epitope spreading occurs early in the disease process and is associated with increased disease severity (25–28). Mechanistically, one can easily envision that, once initiated with one antigen, for example insulin or GAD in the case of T1D – chronic inflammation and local activation of APCs will lead to presentation of more autoantigens, either in their native form or also altered, for example by disulfide bond formation, deamidation, citrullination, or phosphorylation (47). It is also known that individuals with autoantibodies to more than one autoantigen have a higher risk of progression to clinical disease. This has led to the widely held assumption that immune responses to all of these autoantigens and neo-antigens must contribute to disease pathogenesis (i.e. beta cell destruction). But do they? Lessons from animal models actually paint a different and more diverging picture here: Several models show that once an autoantigen such as insulin is in the ‘driver’s seat’ it remains there, regardless of whether other autoantigens also become targets (48, 49). In these models, while new autoantigens become a target of autoimmune responses, these newly formed responses do not accelerate the development of clinical disease in a tangible/relevant way. This insight is therapeutically relevant: If in a given patient, we suspect one autoantigen to be the driving antigen, we might only need to induce tolerance to this one antigen. This would make the daunting task of inducing antigenic tolerance a bit easier but would require identification of the original antigenic stimulus. A counterargument here is that – at least in its early stages – T1D appears to evolve in a relapsing-remitting fashion (29), as we often see no insulitis in pancreata of autoantibody positive organ donors. Therefore, it might be that each flare of islet destruction could be driven by different antigens, as it was for the case of the experimental autoimmune encephalomyelitis model for MS. We will only be able to resolve this puzzle in humans once we know how to induce antigen specific tolerance.

### 2.5 Persistent infections

Persistent infections are caused by pathogens that are able to evade or suppress the immune system and establish a long-term infection and they are thought to be one of several potential mechanisms that can lead to autoimmunity (30).

One example of this is the link between chronic hepatitis C virus (HCV) infection and the development of autoimmune disorders, such as cryoglobulinemia and autoimmune hepatitis (50). The persistent presence of HCV in the liver can trigger an immune response that targets not only the virus, but also the liver cells. This results in chronic inflammation and the production of autoantibodies that cause liver damage and autoimmune disease (51). Another example is the association between chronic infections with the bacterium *Helicobacter pylori* and autoimmune diseases, such as autoimmune gastritis and gastric cancer. The persistent presence of *H. pylori* in the stomach can trigger an immune response that targets both the bacteria and the gastric cells, resulting in chronic inflammation and the production of autoantibodies (52). In addition to such molecular mimicry, persistent infections can also contribute to the development of autoimmunity by inducing polyclonal B cell activation, leading to the production of autoantibodies. For example, chronic infection with EBV has been implicated in the development of SLE, where the virus can induce polyclonal B cell activation and the production of autoantibodies (53). Furthermore, persistent infections can also lead to the activation of Toll-like receptors (TLR), which are involved in the recognition of pathogen-associated molecular patterns. TLR activation can trigger the production of pro-inflammatory cytokines and chemokines, leading to chronic inflammation and tissue damage, and potentially contributing to the development of autoimmune diseases (54).

The emerging picture regarding the association between viral infections and T1D is, that it’s not likely that only one single virus is responsible for T1D acceleration. Rather, various viruses which are found at higher frequency in the pancreata from T1D patients (such as HSV 6)(55), are responsible. This leads us to believe that perhaps the pancreas from potential individuals with T1D might be more susceptible to viral infections in general, which then worsen autoimmunity and beta cell destruction. A meta-analysis has demonstrated that the presence of multiple virus-positive samples amplifies the risk of islet autoimmunity in early childhood, which gives further evidence for a potential role for viral persistence or prolonged infection in the development of T1D. Specifically, consecutive, or prolonged shedding of EV is strongly linked to autoimmune responses in the islets (31).

Nekoua et al. (32) previously discussed the hypothesis that viruses, particularly enteroviruses, may persist in patients with T1D. This persistence is indicated by the recurring detection of enteroviral RNA in leukocytes and mononuclear cells, rather than in serum or plasma. Additionally, in both new-onset and existing T1D, CD14+ monocytes are often found to contain enterovirus RNA. This finding supports the hypothesis that enteroviruses could remain in these immune cells beyond the initial infection phase, potentially influencing the development and course of T1D. The authors also propose that the persistence of Coxsackievirus B (CVB) in various sites, including the gut, blood cells, and the thymus, could act as a reservoir for infection or reinfection of the pancreas. This may result in a disturbance of central tolerance, potentially leading to islet autoimmunity and the development of T1D.

### 2.6 Mechanisms of viral-induced insulin resistance

**Figure 1:**
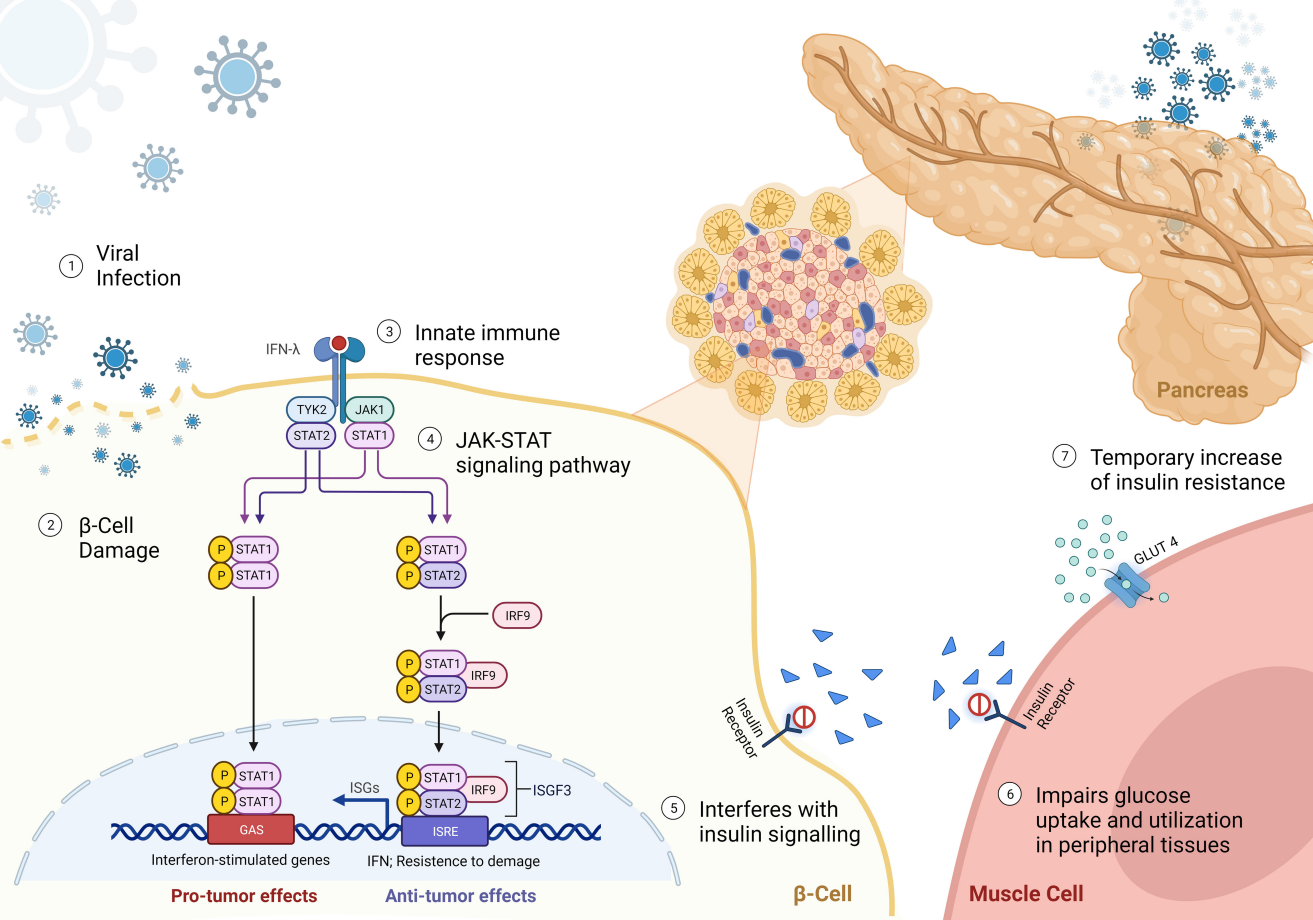
Mechanisms of viral-induced insulin resistance - (1) Virus approaching beta-cell; (2) beta-cell direct damage by viral infection; (3-4) Binding of interferon-γ (IFNγ) to the receptor leads the activation of Janus kinase 1 (JAK1) and JAK2 to undergo autophosphorylation, and to phosphorylate both IFNGR subunits. This leads to recruitment of signal transducer and activator of transcription 1 (STAT1), which binds to IFNGR1 and is phosphorylated in the C terminus by JAK1 and JAK2. Phosphorylated STAT1 monomers form homodimers and translocate to the nucleus, where they bind to IFNγ-activation site (GAS) elements in IFN-stimulated genes (ISG) and activate their transcription. IFNγ exerts multiple effects on immune responses, including innate immunity through its effects on monocytes and macrophages, adaptive immunity through its effects on T cells and B cells, and the inflammatory process through its effects on non-immune cell type. (5) reduced binding of insulin to receptor; and (6) impairment of glucose uptake by peripheral tissues, leading to a reduced translocation of GLUT4 to the cell membrane. (7) Resulting in a temporary hyperglycemia caused by insulin resistance.

In addition to direct beta cell damage, viral infections can induce insulin resistance through the release of interferons, primarily type I interferons (IFN-α and IFN-β) (56) (**Figure 1**). Interferons are key mediators of the antiviral immune response and play a critical role in host defence to viral infections. However, their sustained production during chronic viral infections or repeated acute infections can lead to insulin resistance. Interferons activate the JAK-STAT signalling pathway, which interferes with insulin signalling and impairs glucose uptake and utilization in peripheral tissues, including skeletal muscle, liver, and adipose tissue (33). In addition, even temporary increase of insulin resistance will exert additional stress on beta cells by increasing demand. The “accelerator hypothesis” was previously discussed and suggests that increased body weight raises the demand for insulin, stressing beta cells and making them more susceptible to autoimmune attacks (34). Obesity-related ectopic lipid deposition in islets also triggers beta cell apoptosis, contributing to T1D onset. Furthermore, adipose tissue produces adipokines that generate reactive oxygen species and pro-inflammatory molecules, leading to a dysfunctional antioxidant system and insulin resistance (35, 36). This can increase stress and thus indirectly increase the risk for augmenting beta cell autoimmunity. Furthermore, there is a higher likelihood that the beta cell mass will suddenly become insufficient, thus precipitating clinical diagnosis of diabetes (57). Consequently, this effect can precipitate the development of both T1D and T2D.

## 3 COVID-19

The relationship between coronavirus disease 2019 (COVID-19; caused by the SARS-CoV-2 virus) and autoimmunity is still being studied, but there is some evidence to suggest that COVID-19 may trigger autoimmune reactions in some people. However, recently a large-scale pediatric study conducted in the TEDDY cohort did not show any enhancement of autoimmunity following SARS-CoV-2 infection (58). Thus, the situation remains unclear.

One theory is that SARS-CoV-2infection that causes – in some cases - an overactive immune response, can lead to tissue damage through inflammation. As coronaviruses can infect the pancreas as well, this might accelerate diabetes development in some cases (59). Indeed, there is some evidence that the global pandemic nature of COVID-19 may have caused an increased incidence of T1D. A meta-analysis of 38,149 newly diagnosed cases of T1D in children and adolescents found that the incidence rate was 1.14 times higher in the first year and 1.27 times higher in the second year after the onset of the COVID-19 pandemic compared with before the pandemic (60). SARS-CoV-2 infection can also trigger the upregulation of interferon-induced transmembrane proteins in human pancreatic islets. Researchers observed the expression of ACE2 and TMPRSS2, which are known entry factors for SARS-CoV-2, confirming its infection and replication in human pancreatic islet cells (61).

The second mechanism which may be responsible for the increase in cases of T1D following SARS-CoV-2 infection is an increased demand/pressure on beta cells by a temporary increase of insulin resistance during infection (62). Finally, the third mechanism which may be responsible is direct damage of beta cells by SARS-CoV-2 infection. In this case direct infection of selective cells would lead to their functional impairment and possible demise, as part of the immune system’s task to eliminate infected cells. There is evidence that SARS-CoV-2may trigger or exacerbate autoimmune disease overall in some individuals. SARS-CoV-2exhibits similarities to autoimmune diseases in terms of clinical manifestations, immune responses, and pathogenic mechanisms. There is speculation that SARS-CoV-2 may disrupt self-tolerance and incite autoimmune reactions by revealing host cells to the immune system or, maybe, by exhibiting cross-reactivity with host cells. (63–65).

To date, the relationship between the COVID-19 pandemic and autoimmunity is complex and not fully understood. More research is needed to understand the mechanisms by which COVID-19 may affect the development and progression of autoimmune diseases.

## 4 Herpesviruses

Infection with herpesviruses, in particular beta-herpesviruses, has been associated with the development of autoimmunity, including T1D (66). After primary infection, beta-herpesviruses, like other herpesvirus, enter a latent state within a wide range of cells and tissues. Subsequent reactivation of the infection can occur spontaneously, especially in immunosuppressed people. One of the most ubiquitous beta-herpesviruses is human herpesvirus-6 (HHV-6) that causes roseola infantum, which in itself is a relatively harmless childhood disease (67). However, HHV-6 infection has been implicated in the development of several autoimmune disorders (68). The virus replicates predominantly in T cells and may itself cause immunosuppression. Owing to its broad tropism, HHV-6 has been shown to be present in multiple tissues, including in major organs such as the thyroid and kidney as well as in the liver (69). The correlation of the presence of HHV-6 with the development of T1D was recently examined. Whilst HHV-6 DNA was detected in all donors regardless of diabetes status, it was shown that the presence of HHV-6 gB protein was more frequent in the pancreatic islets and exocrine tissue of those with T1D than those without T1D (55). The conclusion from the studies was that although there was increased frequency of infection of HHV-6 in pancreata from patients with T1D, there was not a direct association with islet pathology. These findings should prompt further studies to understand why certain viral infections occur more frequently in the diabetic pancreas and how they might trigger and/or aid in the development of the disease. In addition, this study among others (70) provides more evidence for the hypothesis that the T1D pancreas appears more susceptible to viral infections and that as such, local infections would impair islet function. However, the enigma that remains unsolved is the lack of a consistent link between the presence of viral antigens and the occurence of insulitis on a per-islet basis (71).

## 5 Enteroviruses

As a major human pathogen, enteroviruses (EV) are common small RNA viruses of the *Picornaviridae* family. Around 100 different types of EV are classified into groups A-D, including polio-, and echoviruses and the numbered EV. Coxsackieviruses are now distributed across three different enterovirus species (EV A, EV B, and EV C).

EV have long been positioned as a potential major viral culprit in the etiology of T1D. However, decades of investigations and compelling evidence have not fully confirmed the connection and studies have provided somewhat conflicting results and conclusions (25). Individuals with T1D from the Diabetes Virus Detection Study (DiViD) had EV in their pancreata, while only two of the eleven individuals without diabetes showed the presence of the virus. Other viral agents were not found in the pancreata of the patients with T1D (72). A recent meta-analysis showed a significant association between EV and pancreatic islet autoimmunity, T1D, or the onset of T1D within one month (73). This study further revealed that the detection of multiple or consecutive EV infections is correlated with islet autoimmunity,specifically, EV-B was strongly linked to T1D (73). Another metanalysis also provided strong evidence, showing a statistically significant association between EV and the development of autoimmunity, including T1D (74). Other evidence in support of the association includes epidemiological data suggesting increased occurrence of T1D amongst relatives with EV infection, a seasonal pattern of T1D development suggesting an increased incidence of T1D following periods with high presence of EV in communities, and high EV antibody titers during pregnancy in women giving birth to children who were later were diagnosed with T1D (75). Furthermore, it is possible that there is an association between persistent or ongoing EV gut infections and the development of T1D (76). Recently, the DiViD team in Oslo published a study that demonstrated preservation of beta cell function (analyzed through c-peptide levels) following antiviral therapy (pleconaril and ribavirin) after the diagnosis of T1D (77). Notably, this effect was observed only after 12 months of treatment and not within the initial 6 months. While the exact reasons for this discrepancy remain unclear, it is evident that further research will be necessary. However, this significant study represents the first piece of evidence supporting the clinical significance of infections in accelerating the decline of beta cells.

Thus, whilst an association between infection with EV and the development of autoimmunity now appears to be well-founded, the exact pathogenic mechanisms remain less well understood. EV are known to interact with a variety of cell-surface receptors to enter cells during infection in humans, and the coxsackie and adenovirus receptor (CAR) has been implicated for T1D because the CAR is expressed by pancreatic alpha and beta cells, in which EV are able to replicate (78). EV infection of beta cells (acute as well as persistent) has been shown to negatively impact their function and even cause cell death or dysfunction, leading to diminished insulin production and secretion (79–83). In sudden-onset T1D, EV infection affects both the endocrine and exocrine pancreas, leading to distinct pathological changes. Within the islet cells, EV infection and replication is accompanied by the expression of 2A protease, which induces chronic inflammation in the islets over time, whereas in the exocrine pancreas, EV infection causes inflammation, changes in the pancreatic structure and fibrosis (84). Indeed, recent findings showed that beta cells infected by coxsackievirus-B (CVB) virus, were more effectively killed by CVB itself than by CVB-reactive T cells, suggesting limited T-cell responses to CVB and supporting the potential for CVB vaccination trials as a strategy for preventing T1D (85).

It also remains unclear, whether EV infections accelerate T1D development as ‘hit and run’ events (meaning they do not persist but aggravate or set-off a deleterious auto-inflammatory cascade by directly and acutely infecting the pancreas) or whether they can persist longer under certain circumstances, for example if there were defects in the interferon systems of diabetes susceptible individuals (10, 78). Interestingly, the company ProventionBio (now acquired by Sanofi) is developing a vaccine for certain human EV strains thought to be responsible for accelerating the development of T1D. Trials with such a vaccine would show whether some EV strains commonly cause T1D.

## 6 Rotaviruses

Consisting of 11 double-stranded RNA segments, there are nine species of rotavirus, however rotavirus A accounts for more than 90% of rotaviral infection in humans (86). Rotaviral infections constitute one of the major reasons for mortality in developing countries and are a leading cause of acute gastroenteritis in children across the world. Rotaviral infection is therefore often mainly located in intestinal cells, giving rise to enteritis and other symptoms such as convulsions (87). However, studies have found wide-spread systemic localization of rotavirus infections in tissues and organs such as the liver, pancreas, and nervous system (88, 89).

One of the major ways that rotaviruses induce autoimmunity is by molecular mimicry owing to sequence resemblance across rotaviral proteins and human antigens (90). For example, rotavirus protein VP7 shares homology with pancreatic islet antigens IA-2 and GAD65, which are central in the pathophysiology of T1D. Other rotavirus proteins have been implicated via molecular mimicry in other autoimmune diseases, including celiac disease (91) and myasthenia gravis (92). In T1D, studies have found that susceptibility to molecular mimicry is likely based on genetic factors, with HLA variants playing a decisive role (8). Specifically, those carrying the HLA-DRB1*04 allele appeared to be most at risk, in line with other findings implicating this HLA variant in the overall susceptibility towards developing T1D (93, 94). In addition, preliminary evidence supports the hypothesis that inhibition of MHCI expression may be important for immune evasion by rotavirus (95).

## 7 Other viruses

Research on the infectious causes of islet autoimmunity in T1D has primarily focused on viral infections in the gut and pancreas. However, respiratory tract infections, particularly within the first year of life, have also been investigated as potential risk factors for childhood T1D. Lower respiratory tract infections (RTI: such as pneumonia and bronchitis) and upper RTI (including rhinitis and pharyngitis) have been examined. Some retrospective studies have shown a significant association between RTI and T1D (96).

Another common infection, EBV has been shown to be the cause of several autoimmune diseases, besides cancer, and studies have shown that EBV-infected individuals have a higher frequency of autoimmune disease, including SLE, RA, and SS, compared to non-infected individuals (97). EBV also plays a critical causal role in MS, especially in genetically susceptible individuals (98). However, there is no evidence of an association between EBV infection and T1D. Moreover, the TEDDY study found that both gastrointestinal infections and Norwalk viruses were associated with an increased risk of insulin autoantibody (IAA) positivity when reported before a child reached one year of age. Conversely, gastrointestinal infections reported during the second year of life were linked to a decreased risk of IAA development (99). These findings support the discussion that early infections may potentially hasten the onset of islet autoimmunity. See **Table 2** for an overview of viral infections and their potential mechanisms in the pathogenesis of T1D.

**Table 2:** Viral Infections and Their Potential Mechanisms in the Pathogenesis of Type 1 Diabetes.

Virus	Potential Proposed Mechanisms in T1DM Pathogenesis
Human Enterovirus A (e.g., Coxsackievirus A4, A2, A16, Enterovirus A71)	Induction of autoimmunity through molecular mimicry or bystander activation. Potential direct beta-cell damage. Epitope spreading to neoantigens. Persistent infections possibly leading to continuous immune stimulation.
Human Enterovirus B (e.g., Echovirus, Coxsackievirus B)	Potential for direct cytopathic effect on beta cells. Triggering of autoimmunity through molecular mimicry. Bystander activation and epitope spreading. Persistent infections contributing to ongoing immune response. Molecular mimicry leading to autoimmune response against beta cells. Direct cytopathic effects on beta cells. Persistent infections contributing to chronic immune activation.
Rubella Virus	Congenital infection leading to increased risk of autoimmune diseases, including T1DM; molecular mimicry. Possible involvement in bystander activation. Epitope spreading in the context of congenital infection.
Cytomegalovirus (CMV)	Possible induction of autoimmunity. CMV infection in beta cells could trigger an immune response leading to beta-cell destruction. Potential for persistent infections affecting immune regulation. Possible role in viral-induced insulin resistance.
Mumps Virus	Historically linked to beta-cell damage and autoimmunity, but current evidence is limited. Possible molecular mimicry and bystander activation. Potential for persistent infection impacting immune responses.
Rotavirus	Potential acceleration of autoimmunity in genetically predisposed individuals. Molecular mimicry and immune system activation. Bystander activation leading to autoimmunity. Epitope spreading following acute infection.
Human Herpesvirus 6 (HHV-6)	Less clear mechanism; possible indirect role through immune system modulation. Direct infection of pancreatic cells. Possible role in bystander activation and epitope spreading. HHV-6-induced insulin resistance is speculative but worth exploring.
Parvovirus B19	Potential indirect role through immune system dysregulation. Some evidence of molecular mimicry. Possible involvement in bystander activation and epitope spreading. Parvovirus B19's role in insulin resistance is not well established but could be investigated.
SARS-CoV-2	Potential triggering of autoimmune reactions. Overactive immune response leading to tissue damage and possibly accelerating diabetes development. Increased incidence of T1D observed during the COVID-19 pandemic. Direct beta-cell damage and upregulation of pancreatic islet cell proteins facilitating viral entry and replication. Temporary increase in insulin resistance during infection. Potential disruption of self-tolerance and autoimmune reactions through molecular mimicry or immune system exposure to host cells.
Herpesviruses (e.g., HHV-6)	Association with autoimmunity, including T1DM. Latent infection with reactivation potential, especially in immunosuppressed individuals. Predominant replication in T cells, potentially causing immunosuppression. Presence in various tissues including pancreatic islets; more frequent in T1DM pancreata but no direct association with islet pathology. Increased susceptibility of T1DM pancreas to viral infections.

## 8 Perspectives and considerations

### 8.1 Revisiting the hygiene hypothesis in T1D etiology

The hygiene hypothesis has gained significant attention in recent years as a potential explanation for the rising prevalence of autoimmune diseases, including T1D. This hypothesis posits that the absence of early-life infections and exposure to diverse microorganisms can alter immune development and increase the risk of developing autoimmune diseases, such as T1D (100, 101). The hypothesis suggests that exposure to a variety of microorganisms, including harmless or beneficial ones, is required to keep the immune system functional, thus reducing the likelihood of autoimmune reactions. Improved hygiene practices and the advent of vaccines have significantly reduced the incidence of various infectious diseases, decreasing the population immunity. This phenomenon presents an “epidemiological paradox.” In environments with lower exposure to virus such as enteroviruses and consequently lower population immunity, there might be a higher risk of T1D due to more severe, late-life infections that can trigger an autoimmune response against pancreatic cells (102). While this has undeniably contributed to better public health outcomes, it has also resulted in decreased exposure to certain pathogens during critical periods of immune system development. This reduction in microbial diversity and antigenic stimulation may have unintended consequences for immune tolerance and regulation. Reduced exposure to infectious agents during early childhood due to improved hygiene practices and reduced pathogen burden may contribute to the development of immune dysregulation and autoimmune disorders. While often dangerous and clearly deleterious, the historical exposure to viral infections during early childhood has been hypothesized to shape the immune system’s maturation and prevent the development of autoimmune responses. Thus, by avoiding mortality from childhood infections through vaccination and better hygiene, we might have increased the risk for autoimmunity, still a decent trade-off, if one considers the amazing reduction in mortality over the past century.

What are the fundamental mechanisms underlying the tuning of the immune system by infections? This question holds significant importance, as gaining insights into it could potentially unveil novel therapeutic avenues. A pivotal mechanism undoubtedly revolves around how each infection, particularly the more substantial ones, triggers the inherent self-regulatory pathways within the immune system. This orchestration aims to curtail immune-driven damage, with every immune response being inherently governed by substantial counter-regulatory mechanisms. It is easy to envision that such mechanisms will also reduce autoimmune responses and their emergence, as bystanders. For example, apoptosis of activated T-cells by TNF and other factors could well be important in such a situation (103). Furthermore, counter-regulatory molecules up-regulated during immune activation (CTLA-4 and PD1-L) are essential. Lastly, some studies have shown that abrogation of diabetes by viral infection that is commonly seen in animal models is accompanied by a systemic increase of immune regulatory cells (104).

### 8.2 Viral infections and therapeutic innovations in T1D

As our understanding of the multifaceted role of viral infections in T1D deepens, the focus on developing targeted therapeutic interventions is intensifying. Considering the dual role of viral infections—both as potential triggers and accelerators of autoimmunity—the question arises: Can targeted antiviral interventions serve as an effective preventative or therapeutic measure for T1D?

Recent advancements in immunotherapy and antiviral medications open the door for novel approaches to halt or reverse the autoimmune process (105–107). For instance, monoclonal antibodies targeting specific viral proteins could potentially inhibit the virus’s ability to accelerate autoimmunity (108). Additionally, small molecule inhibitors that interfere with viral replication may offer another avenue for intervention (109). Moreover, advances in personalized medicine allow for the possibility of tailoring antiviral treatments based on an individual’s genetic susceptibility to T1D and their specific viral exposure history (110). Such precision therapy could maximize therapeutic efficacy while minimizing adverse effects. However, the ethical considerations associated with such interventions should not be overlooked. The long-term safety of these approaches, particularly in pediatric populations most at risk for developing T1D, remains uncertain. Moreover, the impact of antiviral interventions on the broader microbial ecology of the host, and its implications for immune system development and function, warrant thorough investigation.

Given the increasing incidence of T1D globally and the complexity of its etiological factors, integrated approaches that combine antiviral treatments with immune modulators may offer the most promise. As we forge ahead in this promising yet challenging landscape, multidisciplinary collaborations that bring together expertise in virology, immunology, endocrinology, and bioinformatics will be pivotal in translating these scientific advancements into tangible therapeutic solutions.

### 8.3 Viral interactions with beta cells: Direct impact and immune responses

Persistent viruses, such as herpesviruses, and certain strains of EV can also have a direct tropism for beta cells (111). The presence of viral particles and viral components would then trigger a cascade of events within beta cells that can lead to dysfunction and even apoptosis. Persistent viral infections in beta cells can induce endoplasmic reticulum stress, causing an imbalance between protein folding demand and capacity (112, 113). This leads to the accumulation of misfolded proteins, activation of unfolded protein response (UPR), and ultimately beta cell apoptosis. Moreover, viral infections can trigger the release of pro-inflammatory cytokines, such as interleukin-1 beta (IL-1β) and TNF, which further contribute to beta cell damage and dysfunction (114, 115).

An interesting question for T1D in the context of viral infections is, whether an APC presenting viral antigens and becoming activated in response to a local viral infection could then also present autoantigens such as pre-proinsulin and help to activate pre-existing yet usually resting autoreactive cells? There is evidence to show that T cells from individuals with T1D recognize not only the beta cell antigens, but also other self-antigens such as heat shock proteins and other autoantigens (116). Is there evidence that APC licensing can be induced by environmental factors such as toxins, drugs, or stress, which can cause tissue damage and inflammation, leading to the release of self-antigens that can activate T cells? It is a possible hypothesis but conclusively proving this is difficult, as it would necessitate the isolation of APC that present foreign and autoantigens at the same time and requires a yet un-precedented understanding of the local immune dynamics in human islets and draining lymph nodes. However, new technologies for example using living organ donor slices, might be able to further our insight into this potential mechanism (117). In the context of these intricate immune dynamics within pancreatic islets and the potential activation of autoreactive cells by antigen-presenting cells, it becomes evident that beta cell antigen-specific interventions have been devised (118, 119). These interventions hold promise in targeting inflammatory lymphocytes, inducing apoptosis, or inhibiting their trafficking to pancreatic islets, presenting a promising avenue for patients that could potentially sidestep undesirable side effects.

### 8.4 Vaccinations and antiviral treatment for pioneering prevention of autoimmunity

How can we understand the clinical importance of viral infections in the development of T1D? We look forward to several key clinical investigations that will shine more light on this complex situation: Recently, the findings from a study centered around administering antiviral medicationsto individuals recently diagnosed with T1D was published (120, 121). This investigation aimed to determine whether the antiviral potency of these medications is sufficiently high enough. It sought to ascertain whether the heightened prevalence of viruses within the pancreata of patients with T1D can expedite the loss of beta cells post-diagnosis. Indeed, in DiViD study C-peptide was preserved more efficaciously in patients receiving antiviral compounds (pleconaril and ribavirin) compared to those receiving control treatment, demonstrating a preliminary proof for the viability of the proposed antiviral strategies (77)These insights in a small cohort could pave the way for forthcoming clinical trials focused on preventive measures.

In investigating the T1D progression, some trials have also demonstrated that immunotherapies can modulate the advancement of the disease, with evidence that dual courses of teplizumab treatment contribute to the preservation of C-peptide levels two years subsequent to the initial therapy in patients with new-onset T1D (122).

Peering into the future, the spotlight falls on the enteroviral vaccine, which is being tested in some pre-clinical as dicussed by Alhazmi et al. (123): a study on a formalin-inactivated, non-adjuvanted CVB1 vaccine showed it to be highly effective, generating strong neutralizing antibodies and protecting against both acute CVB1 infection in NOD mice and CVB1-induced diabetes in SOCS1-tg mice. Additionally, a hexavalent vaccine containing formalin-inactivated CVB1–6 showed promising results, being safe and effective in producing strong neutralizing antibodies in both mice and rhesus macaques. It prevented CVB-induced myocarditis and diabetes in mice. Safety and efficacy in adults are being assessed in a clinical trial (NCT04690426).

Due to concerns about the safety of live attenuated and formalin-inactivated vaccines, newer vaccine models like virus-like particle vaccines are being developed in collaboration between ProventionBio and Sanofi. These represent a safer and potentially effective alternative to combat CVB infections andwill determine whether specific strains of EV act either as a cause or accelerator in the development of T1D. Further down the path perhaps we can aspire to have the ability to selectively induce tolerance towards viral epitopes that might exhibit cross-reactivity with islet determinants. Regrettably, as of now, this remains an aspiration for future studies to realize.

## Author contributions

JL: Writing – original draft, Writing – review & editing. KH: Writing – review & editing. MH: Writing – review & editing, Writing – original draft.

## References

[1] Houeiss P Luce S Boitard C. Environmental triggering of type 1 diabetes autoimmunity. Front Endocrinol (Lausanne) (2022) 13:933965. doi: 10.3389/fendo.2022.933965 35937815 PMC9353023

[2] Luckett AM Weedon MN Hawkes G Leslie RD Oram RA Grant SFA. Utility of genetic risk scores in type 1 diabetes. Diabetologia (2023) 66(9):1589–600. doi: 10.1007/s00125-023-05955-y PMC10390619 37439792

[3] Aarnisalo J Veijola R Vainionpää R Simell O Knip M Ilonen J. Cytomegalovirus infection in early infancy: risk of induction and progression of autoimmunity associated with type 1 diabetes. Diabetologia (2008) 51:769–72. doi: 10.1007/s00125-008-0945-8 18278478

[4] Oikarinen S Tauriainen S Hober D Lucas B Vazeou A Sioofy-Khojine A. Virus antibody survey in different European populations indicates risk association between Coxsackievirus B1 and type 1 diabetes. Diabetes (2014) 63:655–62. doi: 10.2337/db13-0620 24009257

[5] Alnek K Talja I Laht B Metsküla K Mandel M Reppo I. Iga-type enterovirus antibodies are increased among adults and children with recently diagnosed type 1 diabetes. BioMed Res Int (2022) 2022:7603062. doi: 10.1155/2022/7603062 35958821 PMC9357813

[6] Lampousi AM Carlsson S Löfvenborg JE. Dietary factors and risk of islet autoimmunity and type 1 diabetes: A systematic review and meta-analysis. Ebiomedicine (2021) 72:103633. doi: 10.1016/j.ebiom.2021.103633 34656932 PMC8523874

[7] Bender C Rajendran S Von Herrath MG. New insights into the role of autoreactive Cd8 T cells and cytokines in human type 1 diabetes. Front Endocrinol (Lausanne) (2020) 11:606434. doi: 10.3389/fendo.2020.606434 33469446 PMC7813992

[8] Cusick MF Libbey JE Fujinami RS. Molecular mimicry as A mechanism of autoimmune disease. Clin Rev Allergy Immunol (2012) 42:102–11. doi: 10.1007/s12016-011-8294-7 PMC3266166 22095454

[9] Shim CH Cho S Shin YM Choi JM. Emerging role of bystander T cell activation in autoimmune diseases. Bmb Rep (2022) 55:57–64. doi: 10.5483/BMBRep.2022.55.2.183 35000675 PMC8891623

[10] Apaolaza PS Balcacean D Zapardiel-Gonzalo J Nelson G Lenchik N Akhbari P. Islet expression of type I interferon response sensors is associated with immune infiltration and viral infection in type 1 diabetes. Sci Adv (2021) 7(9):eabd6527. doi: 10.1126/sciadv.abd6527 33627420 PMC7904254

[11] Wenzlau JM Gu Y Michels A Rewers M Haskins K Yu L. Identification of autoantibodies to A hybrid insulin peptide in type 1 diabetes. Diagnostics (Basel) (2023) 13(17):2859. doi: 10.3390/diagnostics13172859 37685398 PMC10487141

[12] Shichkin VP Antica M. Key factors for thymic function and development. Front Immunol (2022) 13:926516. doi: 10.3389/fimmu.2022.926516 35844535 PMC9280625

[13] Yu H Paiva R Flavell RA. Harnessing the power of regulatory T-cells to control autoimmune diabetes: overview and perspective. Immunology (2018) 153:161–70. doi: 10.1111/imm.12867 PMC5765377 29155454

[14] Castañeda J Hidalgo Y Sauma D Rosemblatt M Bono MR Núñez S. The multifaceted roles of B cells in the thymus: from immune tolerance to autoimmunity. Front Immunol (2021) 12:766698. doi: 10.3389/fimmu.2021.766698 34790201 PMC8591215

[15] Sharma A Rudra D. Emerging functions of regulatory T cells in tissue homeostasis. Front Immunol (2018) 9:883. doi: 10.3389/fimmu.2018.00883 29887862 PMC5989423

[16] Ihantola EL Viisanen T Gazali AM Näntö-Salonen K Juutilainen A Moilanen L. Effector T cell resistance to suppression and Stat3 signaling during the development of human type 1 diabetes. J Immunol (2018) 201:1144–53. doi: 10.4049/jimmunol.1701199 30006377

[17] Rodriguez-Calvo T Christoffersson G Bender C Von Herrath MG Mallone R Kent SC. Means, motive, and opportunity: do non-islet-reactive infiltrating T cells contribute to autoimmunity in type 1 diabetes? Front Immunol (2021) 12:683091. doi: 10.3389/fimmu.2021.683091 34220832 PMC8242234

[18] Fujinami RS Oldstone MB Wroblewska Z Frankel ME Koprowski H. Molecular mimicry in virus infection: crossreaction of measles virus phosphoprotein or of herpes simplex virus protein with human intermediate filaments. Proc Natl Acad Sci U.S.A. (1983) 80:2346–50. doi: 10.1073/pnas.80.8.2346 PMC393817 6300911

[19] Gauntt CJ Arizpe HM Higdon AL Wood HJ Bowers DF Rozek MM. Molecular mimicry, anti-Coxsackievirus B3 neutralizing monoclonal antibodies, and myocarditis. J Immunol (1995) 154:2983–95. doi: 10.4049/jimmunol.154.6.2983 7533190

[20] Vreugdenhil GR Geluk A Ottenhoff TH Melchers WJ Roep BO Galama JM. Molecular mimicry in diabetes mellitus: the homologous domain in Coxsackie B virus protein 2c and islet autoantigen Gad65 is highly conserved in the Coxsackie B-like enteroviruses and binds to the diabetes associated Hla-Dr3 molecule. Diabetologia (1998) 41:40–6. doi: 10.1007/s001250050864 9498628

[21] Wucherpfennig KW Strominger JL. Molecular mimicry in T cell-mediated autoimmunity: viral peptides activate human T cell clones specific for myelin basic protein. Cell (1995) 80:695–705. doi: 10.1016/0092-8674(95)90348-8 7534214 PMC7133435

[22] Zhao ZS Granucci F Yeh L Schaffer PA Cantor H. Molecular mimicry by herpes simplex virus-type 1: autoimmune disease after viral infection. Science (1998) 279:1344–7. doi: 10.1126/science.279.5355.1344 9478893

[23] Coppieters KT Wiberg A Von Herrath MG. Viral infections and molecular mimicry in type 1 diabetes. Apmis (2012) 120:941–9. doi: 10.1111/apm.12011 PMC5774991 23051179

[24] Christoffersson G Ratliff SS Von Herrath MG. Interference with pancreatic sympathetic signaling halts the onset of diabetes in mice. Sci Adv (2020) 6(35):eabb2878. doi: 10.1126/sciadv.abb2878 33052874 PMC7531904

[25] Smatti MK Cyprian FS Nasrallah GK Al Thani AA Almishal RO Yassine HM. Viruses and autoimmunity: A review on the potential interaction and molecular mechanisms. Viruses (2019) 11(8):762. doi: 10.3390/v11080762 31430946 PMC6723519

[26] Quintana FJ Patel B Yeste A Nyirenda M Kenison J Rahbari R. Epitope spreading as an early pathogenic event in pediatric multiple sclerosis. Neurology (2014) 83:2219–26. doi: 10.1212/WNL.0000000000001066 PMC4277672 25381299

[27] Miller SD Katz-Levy Y Neville KL Vanderlugt CL. Virus-induced autoimmunity: epitope spreading to myelin autoepitopes in Theiler's virus infection of the central nervous system. Adv Virus Res (2001) 56:199–217. doi: 10.1016/S0065-3527(01)56008-X 11450300

[28] Tuohy VK Kinkel RP. Epitope spreading: A mechanism for progression of autoimmune disease. Arch Immunol Ther Exp (Warsz) (2000) 48:347–51. 11140461

[29] von Herrath M Sanda S Herold K. Type 1 diabetes as A relapsing-remitting disease? Nat Rev Immunol (2007) 7:988–94. doi: 10.1038/nri2192 17982429

[30] Op de Beeck A Eizirik DL. Viral infections in type 1 diabetes mellitus–why the B cells? Nat Rev Endocrinol (2016) 12:263–73. doi: 10.1038/nrendo.2016.30 PMC5348720 27020257

[31] Faulkner CL Luo YX Isaacs S Rawlinson WD Craig ME Kim KW. The virome in early life and childhood and development of islet autoimmunity and type 1 diabetes: A systematic review and meta-analysis of observational studies. Rev Med Virol (2021) 31:1–14. doi: 10.1002/rmv.2209 PMC8518965 33378601

[32] Nekoua MP Alidjinou EK Hober D. Persistent Coxsackievirus B infection and pathogenesis of type 1 diabetes mellitus. Nat Rev Endocrinol (2022) 18:503–16. doi: 10.1038/s41574-022-00688-1 PMC9157043 35650334

[33] McGillicuddy FC Chiquoine EH Hinkle CC Kim RJ Shah R Roche HM. Interferon gamma attenuates insulin signaling, lipid storage, and differentiation in human adipocytes *via* activation of the Jak/Stat pathway. J Biol Chem (2009) 284:31936–44. doi: 10.1074/jbc.M109.061655 PMC2797265 19776010

[34] Wilkin TJ. The convergence of type 1 and type 2 diabetes in childhood: the accelerator hypothesis. Pediatr Diabetes (2012) 13:334–9. doi: 10.1111/j.1399-5448.2011.00831.x 22059423

[35] Subauste A Gianani R Chang AM Plunkett C Pietropaolo SL Zhang YJ. Islet autoimmunity identifies A unique pattern of impaired pancreatic beta-cell function, markedly reduced pancreatic beta cell mass and insulin resistance in clinically diagnosed type 2 diabetes. PloS One (2014) 9:e106537. doi: 10.1371/journal.pone.0106537 25226365 PMC4165581

[36] Brooks-Worrell BM Tjaden AH Edelstein SL Palomino B Utzschneider KM Arslanian S. Islet autoimmunity in adults with impaired glucose tolerance and recently diagnosed, treatment naïve type 2 diabetes in the restoring insulin secretion (Rise) study. Front Immunol (2021) 12:640251. doi: 10.3389/fimmu.2021.640251 33981301 PMC8108986

[37] Yu W Jiang N Ebert PJ Kidd BA Müller S Lund PJ. Clonal deletion prunes but does not eliminate self-specific Aβ Cd8(+) T lymphocytes. Immunity (2015) 42:929–41. doi: 10.1016/j.immuni.2015.05.001 PMC4455602 25992863

[38] Luczyński W Stasiak-Barmuta A Urban R Urban M Florys B Hryszko M. Lower percentages of T regulatory cells in children with type 1 diabetes - preliminary report. Pediatr Endocrinol Diabetes Metab (2009) 15:34–8. 19454187

[39] Zahran AM Elsayh KI Metwalley KA. Regulatory T cells in children with recently diagnosed type 1 diabetes. Indian J Endocrinol Metab (2012) 16:952–7. doi: 10.4103/2230-8210.102998 PMC3510966 23226641

[40] Visperas A Vignali DA. Are regulatory T cells defective in type 1 diabetes and can we fix them? J Immunol (2016) 197:3762–70. doi: 10.4049/jimmunol.1601118 PMC5119643 27815439

[41] Damian RT. Molecular mimicry: antigen sharing by parasite and host and its consequences. Am Nat (1964) 98:129–49. doi: 1086/282313

[42] Ellis NM Li Y Hildebrand W Fischetti VA Cunningham MW. T cell mimicry and epitope specificity of cross-reactive T cell clones from rheumatic heart disease. J Immunol (2005) 175:5448–56. doi: 10.4049/jimmunol.175.8.5448 16210652

[43] Hedström AK. Risk factors for multiple sclerosis in the context of epstein-barr virus infection. Front Immunol (2023) 14:1212676. doi: 10.3389/fimmu.2023.1212676 37554326 PMC10406387

[44] Khosravi-Maharlooei M Madley R Borsotti C Ferreira LMR Sharp RC Brehm MA. Modeling human T1d-associated autoimmune processes. Mol Metab (2022) 56:101417. doi: 10.1016/j.molmet.2021.101417 34902607 PMC8739876

[45] Christen U Hintermann E Holdener M Von Herrath MG. Viral triggers for autoimmunity: is the 'Glass of molecular mimicry' Half full or half empty? J Autoimmun (2010) 34:38–44. doi: 10.1016/j.jaut.2009.08.001 19716269 PMC2819590

[46] Robinson AP Harp CT Noronha A Miller SD. The experimental autoimmune encephalomyelitis (Eae) model of MS: utility for understanding disease pathophysiology and treatment. Handb Clin Neurol (2014) 122:173–89. doi: 10.1016/B978-0-444-52001-2.00008-X PMC3981554 24507518

[47] Amdare N Purcell AW Dilorenzo TP. Noncontiguous T cell epitopes in autoimmune diabetes: from mice to men and back again. J Biol Chem (2021) 297:100827. doi: 10.1016/j.jbc.2021.100827 34044020 PMC8233151

[48] Martinic MM Juedes AE Bresson D Homann D Skak K Huber C. Minimal impact of A *de novo*-expressed beta-cell autoantigen on spontaneous diabetes development in nod mice. Diabetes (2007) 56:1059–68. doi: 10.2337/db05-0062 17395746

[49] Fousteri G Jasinski J Dave A Nakayama M Pagni P Lambolez F. Following the fate of one insulin-reactive Cd4 T cell: conversion into teffs and tregs in the periphery controls diabetes in nod mice. Diabetes (2012) 61:1169–79. doi: 10.2337/db11-0671 PMC3331775 22403296

[50] Tomasiewicz K Pokora-Pachowicz A Kiciak S. Autoimmune reactions in the course of the hepatitis C virus (HCV) infection. Clin Exp Hepatol (2015) 1:39–43. doi: 10.5114/ceh.2015.51804 28856254 PMC5497407

[51] Zampino R Marrone A Restivo L Guerrera B Sellitto A Rinaldi L. Chronic HCV infection and inflammation: clinical impact on hepatic and extra-hepatic manifestations. World J Hepatol (2013) 5:528–40. doi: 10.4254/wjh.v5.i10.528 PMC3812455 24179612

[52] Wang L Cao ZM Zhang LL Dai XC Liu ZJ Zeng YX. Helicobacter pylori and autoimmune diseases: involving multiple systems. Front Immunol (2022) 13:833424. doi: 10.3389/fimmu.2022.833424 35222423 PMC8866759

[53] Jog NR James JA. Epstein barr virus and autoimmune responses in systemic lupus erythematosus. Front Immunol (2020) 11:623944. doi: 10.3389/fimmu.2020.623944 33613559 PMC7886683

[54] Vijay K. Toll-like receptors in immunity and inflammatory diseases: past, present, and future. Int Immunopharmacol (2018) 59:391–412. doi: 10.1016/j.intimp.2018.03.002 29730580 PMC7106078

[55] Sabouri S Benkahla MA Kiosses WB Rodriguez-Calvo T Zapardiel-Gonzalo J Castillo E. Human herpesvirus-6 is present at higher levels in the pancreatic tissues of donors with type 1 diabetes. J Autoimmun (2020) 107:102378. doi: 10.1016/j.jaut.2019.102378 31818546 PMC7237334

[56] Koivisto VA Pelkonen R Cantell K. Effect of interferon on glucose tolerance and insulin sensitivity. Diabetes (1989) 38:641–7. doi: 10.2337/diab.38.5.641 2653935

[57] Vilarrasa N San Jose P Rubio M Lecube A. Obesity in patients with type 1 diabetes: links, risks and management challenges. Diabetes Metab Syndr Obes (2021) 14:2807–27. doi: 10.2147/DMSO.S223618 PMC8232956 34188505

[58] Krischer JP Lernmark Å. Hagopian WA Rewers MJ Mcindoe R Toppari J. Sars-Cov-2 - no increased islet autoimmunity or type 1 diabetes in teens. N Engl J Med (2023) 389:474–5. doi: 10.1056/NEJMc2216477 PMC10481371 37530831

[59] Ben Nasr M D'addio F Montefusco L Usuelli V Loretelli C Rossi A. Indirect and direct effects of Sars-Cov-2 on human pancreatic islets. Diabetes (2022) 71:1579–90. doi: 10.2337/db21-0926 PMC9490452 35499468

[60] D'Souza D Empringham J Pechlivanoglou P Uleryk EM Cohen E Shulman R. Incidence of diabetes in children and adolescents during the Covid-19 pandemic: A systematic review and meta-analysis. JAMA Netw Open (2023) 6:e2321281. doi: 10.1001/jamanetworkopen.2023.21281 37389869 PMC10314307

[61] Müller JA Gros R Conzelmann C Krüger J Merle U Steinhart J. Sars-Cov-2 infects and replicates in cells of the human endocrine and exocrine pancreas. Nat Metab (2021) 3:149–65. doi: 10.1038/s42255-021-00347-1 33536639

[62] Metwally AA Mehta P Johnson BS Nagarjuna A Snyder MP. Covid-19-induced new-onset diabetes: trends and technologies. Diabetes (2021) 70:2733–44. doi: 10.2337/dbi21-0029 PMC8660988 34686519

[63] Knight JS Caricchio R Casanova JL Combes AJ Diamond B Fox SE. The intersection of Covid-19 and autoimmunity. J Clin Invest (2021) 131(24):e154886. doi: 10.1172/JCI154886 34710063 PMC8670833

[64] Liu Y Sawalha AH Lu Q. Covid-19 and autoimmune diseases. Curr Opin Rheumatol (2021) 33:155–62. doi: 10.1097/BOR.0000000000000776 PMC7880581 33332890

[65] Zebardast A Hasanzadeh A Ebrahimian Shiadeh SA Tourani M Yahyapour Y. COVID-19: A trigger of autoimmune diseases. Cell Biol Int (2023) 47:848–58. doi: 10.1002/cbin.11997 36740221

[66] Arcangeletti MC Caselli E. Recent advances in unveiling the role of beta-herpesviruses in autoimmune diseases. Microorganisms (2021) 47(5):848–58. doi: 10.3390/microorganisms9122572 PMC8705016 34946173

[67] Wolz MM Sciallis GF Pittelkow MR. Human herpesviruses 6, 7, and 8 from A dermatologic perspective. Mayo Clin Proc (2012) 87:1004–14. doi: 10.1016/j.mayocp.2012.04.010 PMC3538396 22819486

[68] Broccolo F Fusetti L Ceccherini-Nelli L. Possible role of human herpesvirus 6 as A trigger of autoimmune disease. Scientificworldjournal (2013) 2013:867389. doi: 10.1155/2013/867389 24282390 PMC3825270

[69] Agut H Bonnafous P Gautheret-Dejean A. Laboratory and clinical aspects of human herpesvirus 6 infections. Clin Microbiol Rev (2015) 28:313–35. doi: 10.1128/CMR.00122-14 PMC4402955 25762531

[70] Roep BO Thomaidou S Van Tienhoven R Zaldumbide A. Type 1 diabetes mellitus as A disease of the B-cell (Do not blame the immune system?). Nat Rev Endocrinol (2021) 17:150–61. doi: 10.1038/s41574-020-00443-4 PMC7722981 33293704

[71] Willcox A Richardson SJ Bone AJ Foulis AK Morgan NG. Immunohistochemical analysis of the relationship between islet cell proliferation and the production of the enteroviral capsid protein, Vp1, in the islets of patients with recent-onset type 1 diabetes. Diabetologia (2011) 54:2417–20. doi: 10.1007/s00125-011-2192-7 21597997

[72] Krogvold L Genoni A Puggioni A Campani D Richardson SJ Flaxman CS. Live enteroviruses, but not other viruses, detected in human pancreas at the onset of type 1 diabetes in the divid study. Diabetologia (2022) 65(12):2108–20. doi: 10.1007/s00125-022-05779-2 PMC9630231 35953727

[73] Isaacs SR Roy A Dance B Ward EJ Foskett DB Maxwell AJ. Enteroviruses and risk of islet autoimmunity or type 1 diabetes: systematic review and meta-analysis of controlled observational studies detecting viral nucleic acids and proteins. Lancet Diabetes Endocrinol (2023) 11(8):578–92. doi: 10.1016/S2213-8587(23)00122-5 37390839

[74] Wang K Ye F Chen Y Xu J Zhao Y Wang Y. Association between enterovirus infection and type 1 diabetes risk: A meta-analysis of 38 case-control studies. Front Endocrinol (Lausanne) (2021) 12:706964. doi: 10.3389/fendo.2021.706964 34557158 PMC8453141

[75] Hober D Sane F. Enteroviral pathogenesis of type 1 diabetes. Discovery Med (2010) 10:151–60. 20807476

[76] Quinn LM Wong FS Narendran P. Environmental determinants of type 1 diabetes: from association to proving causality. Front Immunol (2021) 12:737964. doi: 10.3389/fimmu.2021.737964 34659229 PMC8518604

[77] Krogvold L Mynarek IM Ponzi E Mørk FB Hessel TW Roald T. Pleconaril and Ribavirin in new-onset type 1 diabetes: A phase 2 randomized trial. Nat Med (2023) 29(11):2902–8. doi: 10.1038/s41591-023-02576-1 PMC10667091 37789144

[78] Rodriguez-Calvo T. Enterovirus infection and type 1 diabetes: unraveling the crime scene. Clin Exp Immunol (2019) 195:15–24. doi: 10.1111/cei.13223 30307605 PMC6300647

[79] Petzold A Solimena M Knoch KP. Mechanisms of beta cell dysfunction associated with viral infection. Curr Diabetes Rep (2015) 15:73. doi: 10.1007/s11892-015-0654-x PMC4539350 26280364

[80] Hodik M Skog O Lukinius A Isaza-Correa JM Kuipers J Giepmans BN. Enterovirus infection of human islets of langerhans affects B-cell function resulting in disintegrated islets, decreased glucose stimulated insulin secretion and loss of golgi structure. BMJ Open Diabetes Res Care (2016) 4:e000179. doi: 10.1136/bmjdrc-2015-000179 PMC4985798 27547409

[81] Nyalwidhe JO Gallagher GR Glenn LM Morris MA Vangala P Jurczyk A. Coxsackievirus-induced proteomic alterations in primary human islets provide insights for the etiology of diabetes. J Endocr Soc (2017) 1:1272–86. doi: 10.1210/js.2017-00278 PMC5686651 29264452

[82] Bertin A Sane F Gmyr V Lobert D Dechaumes A Kerr-Conte J. Coxsackievirus-B4 infection of human primary pancreatic ductal cell cultures results in impairment of differentiation into insulin-producing cells. Viruses (2019) 11(7):597. doi: 10.3390/v11070597 31269669 PMC6669621

[83] Nekoua MP Bertin A Sane F Gimeno JP Fournier I Salzet M. Persistence of Coxsackievirus B4 in pancreatic B cells disturbs insulin maturation, pattern of cellular proteins, and Dna methylation. Microorganisms (2021) 9(6):1125. doi: 10.3390/microorganisms9061125 34067388 PMC8224704

[84] Fukui T Kobayashi T Jimbo E Aida K Shimada A Oikawa Y. Bi-glandular and persistent enterovirus infection and distinct changes of the pancreas in slowly progressive type 1 diabetes mellitus. Sci Rep (2023) 13:6977. doi: 10.1038/s41598-023-33011-7 37117225 PMC10147722

[85] Vecchio F Carré A Korenkov D Zhou Z Apaolaza P Tuomela S. Coxsackievirus infection induces direct pancreatic B-cell killing but poor anti-viral Cd8+ T-cell responses. Biorxiv (2023) 21:2023.08.19.553954. doi: 10.1101/2023.08.19.553954 PMC10917340 38446892

[86] Crawford SE Ramani S Tate JE Parashar UD Svensson L Hagbom M. Rotavirus infection. Nat Rev Dis Primers (2017) 3:17083. doi: 10.1038/nrdp.2017.83 29119972 PMC5858916

[87] Hasan H Nasirudeen NA Ruzlan MAF Mohd Jamil MA Ismail NAS Wahab AA. Acute infectious gastroenteritis: the causative agents, omics-based detection of antigens and novel biomarkers. Children (Basel) (2021) 8(12):1112. doi: 10.3390/children8121112 34943308 PMC8700514

[88] Ramig RF. Pathogenesis of intestinal and systemic rotavirus infection. J Virol (2004) 78:10213–20. doi: 10.1128/JVI.78.19.10213-10220.2004 PMC516399 15367586

[89] Dian Z Sun Y Zhang G Xu Y Fan X Yang X. Rotavirus-related systemic diseases: clinical manifestation, evidence and pathogenesis. Crit Rev Microbiol (2021) 47:580–95. doi: 10.1080/1040841X.2021.1907738 33822674

[90] Honeyman MC Stone NL Falk BA Nepom G Harrison LC. Evidence for molecular mimicry between human T cell epitopes in rotavirus and pancreatic islet autoantigens. J Immunol (2010) 184:2204–10. doi: 10.4049/jimmunol.0900709 20083660

[91] Cohen R Mahlab-Guri K Atali M Elbirt D. Viruses and celiac disease: what do we know? Clin Exp Med (2023) 23(7):2931–9. doi: 10.1007/s10238-023-01070-9 PMC10134706 37103650

[92] Sarkar T Das S Nandy P Bhowmick R Nandy A. In silico study of potential autoimmune threats from rotavirus infection. Comput Biol Chem (2014) 51:51–6. doi: 10.1016/j.compbiolchem.2014.05.003 24929545

[93] Pahari S Chatterjee D Negi S Kaur J Singh B Agrewala JN. Morbid sequences suggest molecular mimicry between microbial peptides and self-antigens: A possibility of inciting autoimmunity. Front Microbiol (2017) 8:1938. doi: 10.3389/fmicb.2017.01938 29062305 PMC5640720

[94] Ziegler AG. The countdown to type 1 diabetes: when, how and why does the clock start? Diabetologia (2023) 66:1169–78. doi: 10.1007/s00125-023-05927-2 PMC10212739 37231274

[95] Holloway G Fleming FE Coulson BS. Mhc class I expression in intestinal cells is reduced by rotavirus infection and increased in bystander cells lacking rotavirus antigen. Sci Rep (2018) 8:67. doi: 10.1038/s41598-017-18464-x 29311575 PMC5758578

[96] Wu R Mumtaz M Maxwell AJ Isaacs SR Laiho JE Rawlinson WD. Respiratory infections and type 1 diabetes: potential roles in pathogenesis. Rev Med Virol (2023) 33:E2429. doi: 10.1002/rmv.2429 36790804 PMC10909571

[97] Ascherio A Munger KL. Ebv and autoimmunity. Curr Top Microbiol Immunol (2015) 390:365–85. doi: 10.1007/978-3-319-22822-8_15 26424654

[98] Soldan SS Lieberman PM. Epstein-barr virus and multiple sclerosis. Nat Rev Microbiol (2023) 21:51–64. doi: 10.1038/s41579-022-00770-5 35931816 PMC9362539

[99] Lönnrot M Lynch KF Rewers M Lernmark Å. Vehik K Akolkar B. Gastrointestinal infections modulate the risk for insulin autoantibodies as the first-appearing autoantibody in the teddy study. Diabetes Care (2023) 46(11):1908–15. doi: 10.2337/figshare.23823507.v1 PMC10620548 37607456

[100] Bach JF Chatenoud L. The hygiene hypothesis: an explanation for the increased frequency of insulin-dependent diabetes. Cold Spring Harb Perspect Med (2012) 2:a007799. doi: 10.1101/cshperspect.a007799 22355800 PMC3281594

[101] Murdaca G Greco M Borro M Gangemi S. Hygiene hypothesis and autoimmune diseases: A narrative review of clinical evidences and mechanisms. Autoimmun Rev (2021) 20:102845. doi: 10.1016/j.autrev.2021.102845 33971339

[102] Filippi CM von Herrath MG. Viral trigger for type 1 diabetes: pros and cons. Diabetes (2008) 57:2863–71. doi: 10.2337/db07-1023 PMC2570378 18971433

[103] Nie Z Aboulnasr F Natesampillai S Burke SP Krogman A Bren GD. Both HIV-infected and uninfected cells express trailshort, which confers trail resistance upon bystander cells within the microenvironment. J Immunol (2018) 200:1110–23. doi: 10.4049/jimmunol.1701113 PMC5808399 29263214

[104] Filippi CM Estes EA Oldham JE von Herrath MG. Immunoregulatory mechanisms triggered by viral infections protect from type 1 diabetes in mice. J Clin Invest (2009) 119:1515–23. doi: 10.1172/JCI38503 PMC2689128 19478458

[105] Warshauer JT Bluestone JA Anderson MS. New frontiers in the treatment of type 1 diabetes. Cell Metab (2020) 31:46–61. doi: 10.1016/j.cmet.2019.11.017 31839487 PMC6986815

[106] Bluestone JA Buckner JH Herold KC. Immunotherapy: building A bridge to A cure for type 1 diabetes. Science (2021) 373:510–6. doi: 10.1126/science.abh1654 34326232

[107] Le Coz C Oldridge DA Herati RS De Luna N Garifallou J Cruz Cabrera E. Human T follicular helper clones seed the germinal center-resident regulatory pool. Sci Immunol (2023) 8:eade8162. doi: 10.1126/sciimmunol.ade8162 37027481 PMC10329285

[108] Herold KC Bundy BN Long SA Bluestone JA Dimeglio LA Dufort MJ. An anti-cd3 antibody, teplizumab, in relatives at risk for type 1 diabetes. N Engl J Med (2019) 381:603–13. doi: 10.1056/NEJMoa1902226 PMC6776880 31180194

[109] Nekoua MP Mercier A Alhazmi A Sane F Alidjinou EK Hober D. Fighting enteroviral infections to prevent type 1 diabetes. Microorganisms (2022) 10(4):768. doi: 10.3390/microorganisms10040768 35456818 PMC9031364

[110] Carr ALJ Evans-Molina C Oram RA. Precision medicine in type 1 diabetes. Diabetologia (2022) 65:1854–66. doi: 10.1007/s00125-022-05778-3 PMC9522741 35994083

[111] Smura T Natri O Ylipaasto P Hellman M Al-Hello H Piemonti L. Enterovirus strain and type-specific differences in growth kinetics and virus-induced cell destruction in human pancreatic duct epithelial hpde cells. Virus Res (2015) 210:188–97. doi: 10.1016/j.virusres.2015.08.003 26260332

[112] Bettigole SE Glimcher LH. Endoplasmic reticulum stress in immunity. Annu Rev Immunol (2015) 33:107–38. doi: 10.1146/annurev-immunol-032414-112116 25493331

[113] Oakes SA Papa FR. The role of endoplasmic reticulum stress in human pathology. Annu Rev Pathol (2015) 10:173–94. doi: 10.1146/annurev-pathol-012513-104649 PMC5568783 25387057

[114] Cnop M Welsh N Jonas JC Jörns A Lenzen S Eizirik DL. Mechanisms of pancreatic beta-cell death in type 1 and type 2 diabetes: many differences, few similarities. Diabetes (2005) 54 Suppl 2:S97–107. doi: 10.2337/diabetes.54.suppl_2.s97 16306347

[115] Kany S Vollrath JT Relja B. Cytokines in inflammatory disease. Int J Mol Sci (2019) 20(23):6008. doi: 10.3390/ijms20236008 31795299 PMC6929211

[116] Roep BO Peakman M. Antigen targets of type 1 diabetes autoimmunity. Cold Spring Harb Perspect Med (2012) 2:a007781. doi: 10.1101/cshperspect.a007781 22474615 PMC3312399

[117] Panzer JK Hiller H Cohrs CM Almaça J Enos SJ Beery M. Pancreas tissue slices from organ donors enable *in situ* analysis of type 1 diabetes pathogenesis. JCI Insight (2020) 5(8):e134525. doi: 10.1172/jci.insight.134525 32324170 PMC7205437

[118] Wan X Zaghouani H. Antigen-specific therapy against type 1 diabetes: mechanisms and perspectives. Immunotherapy (2014) 6:155–64. doi: 10.2217/imt.13.172 24491089

[119] Cudini A Fierabracci A. Advances in immunotherapeutic approaches to type 1 diabetes. Int J Mol Sci (2023) 24(11):9220. doi: 10.3390/ijms24119220 37298175 PMC10253167

[120] Dunne JL Richardson SJ Atkinson MA Craig ME Dahl-Jørgensen K Flodström-Tullberg M. Rationale for enteroviral vaccination and antiviral therapies in human type 1 diabetes. Diabetologia (2019) 62:744–53. doi: 10.1007/s00125-019-4811-7 PMC6450860 30675626

[121] Niklasson B Klitz W Juntti-Berggren L Berggren PO Lindquist L. Effectiveness of antivirals in A type 1 diabetes model and the move toward human trials. Viral Immunol (2020) 33:594–9. doi: 10.1089/vim.2020.0039 32758075

[122] Herold KC Gitelman SE Ehlers MR Gottlieb PA Greenbaum CJ Hagopian W. Teplizumab (Anti-cd3 mab) treatment preserves C-peptide responses in patients with new-onset type 1 diabetes in A randomized controlled trial: metabolic and immunologic features at baseline identify A subgroup of responders. Diabetes (2013) 62:3766–74. doi: 10.2337/db13-0345 PMC3806618 23835333

[123] Alhazmi A Nekoua MP Mercier A Vergez I Sane F Alidjinou EK. Combating Coxsackievirus B infections. Rev Med Virol (2023) 33:E2406. doi: 10.1002/rmv.2406 36371612

